# Poly(Vinyl Alcohol)/Poly(Acrylic Acid) Gel Polymer Electrolyte Modified with Multi-Walled Carbon Nanotubes and SiO_2_ Nanospheres to Increase Rechargeability of Zn–Air Batteries

**DOI:** 10.3390/gels10090587

**Published:** 2024-09-12

**Authors:** Lucia Díaz-Patiño, Minerva Guerra-Balcázar, Lorena Álvarez-Contreras, Noé Arjona

**Affiliations:** 1 Centro de Investigación y Desarrollo Tecnológico en Electroquímica, Sanfandila, Pedro Escobedo, Querétaro 76703, Mexico; apatino@cideteq.mx; 2 División de Investigación y Posgrado, Facultad de Ingeniería, Universidad Autónoma de Querétaro, Querétaro 76010, Mexico; minerva.guerra@uaq.mx; 3 Centro de Investigación en Materiales Avanzados S.C., Complejo Industrial Chihuahua, Chihuahua 31136, Mexico

**Keywords:** Zn–air battery, gel polymer electrolyte, polyvinyl alcohol, polyacrylic acid, SiO_2_, carbon nanotubes

## Abstract

Zn–air batteries (ZABs) are a promising technology; however, their commercialization is limited by challenges, including those occurring in the electrolyte, and thus, gel polymer electrolytes (GPEs) and hydrogels have emerged as substitutes for traditional aqueous electrolytes. In this work, PVA/PAA membranes were synthesized by the solvent casting method and soaked in 6 M KOH to act as GPEs. The thickness of the membrane was modified (50, 100, and 150 μm), and after determining the best thickness, the membrane was modified with synthesized SiO_2_ nanospheres and multi-walled carbon nanotubes (CNTs). SEM micrographs revealed that the CNTs displayed lengths of tens of micrometers, having a narrow diameter (95 ± 7 nm). In addition, SEM revealed that the SiO_2_ nanospheres had homogeneous shapes with sizes of 110 ± 10 nm. Physicochemical experiments revealed that SiO_2_ incorporation at 5 wt.% increased the water uptake of the PVA/PAA membrane from 465% to 525% and the ionic conductivity to 170 mS cm^−1^. The further addition of 0.5 wt.% CNTs did not impact the water uptake but it promoted a porous structure, increasing the power density and the stability, showing three-times-higher rechargeability than the ZAB operated with the PVA/PAA GPE.

## 1. Introduction

ZABs have emerged as novel and eco-friendly energy storage systems, and special attention has been given to this technology due to its unique advantages, like high energy density in contrast to Pb-acid- (34 times higher), Ni–metal hydride- (13.5 times higher), and Li-ion batteries (3.4 times higher) [1], and the abundance and low cytotoxicity of Zn metal [2,3]. In addition, Zn reduction (Zn^2+^ + 2e^−^→Zn^0^) is electrochemically favorable, allowing rechargeable ZABs to be produced (RZABs). For the successful implementation of ZABs, major efforts must be made to diminish the main challenges affecting this technology because certain issues have been observed in the three main components of a ZAB: the anode, the air electrode, and the electrolyte.

In a Zn electrode, the contact between the electrode and the electrolyte (typically 6 M KOH) promotes chemical reactions like Zn self-corrosion/dissolution, promoting secondary reactions like the hydrogen evolution reaction (HER) [4]. During discharge, zincate ions are formed, and their accumulation promotes the formation of ZnO, which can be deposited onto the electrode surface, blocking active sites, and thus causing surface passivation. During charge, the uneven deposition of Zn^0^ can promote the formation of dendrites, and it has been observed that these can grow significantly to cause short-circuits [5,6]. In an air electrode, the sluggish kinetics of the oxygen reduction reaction (ORR) taking place during discharge and the oxygen evolution reaction (OER) during charging limit battery performance [7,8]. Consequently, major efforts have been made to develop highly active and durable Pt-free bifunctional electrocatalysts [9,10,11].

The study of electrolytes is fundamental to improving battery performance and rechargeability. The use of 6 M KOH aqueous electrolytes is extensive in ZABs due to their high ionic conductivity; however, as observed in different works [12,13,14], water evaporation requires the continuous refilling of water. In addition, the electrode separation in aqueous ZABs is typically in the order of 1 to 20 mm [15,16], increasing ohmic resistances due to large electrode separation. Thus, new electrolytes should present high ionic conductivity, low thickness (in the order of a few tens of micrometers), excellent mechanical properties, a low tendency to chemically oxidize the Zn electrode, the ability to decrease Zn dendrite formation, and the capability to diminish the HER and the formation of insoluble carbonates from CO_2_ entering from the atmosphere. As observed from these requirements, the formulation of new electrolytes is not an easy task. Nowadays, three groups of electrolytes are being researched: (a) liquid electrolytes (including gels), (b) semi-solid-state electrolytes (like gel polymer electrolytes, GPEs), and (c) all-solid-state electrolytes (like solid polymer electrolytes, SPEs) [17]. 

Gel polymer electrolytes are attractive because, contrary to hydrogels (requiring reservoirs), GPEs can allow the development of flexible ZABs. Furthermore, GPEs can operate in rigid ZABs with the advantages of decreasing electrode separation (decreasing the battery weight and volume), and thus improving the performance. From the literature, it is well known that the most reported polymers for GPEs in ZABs are sodium polyacrylonitrile (PANa), polyvinyl alcohol (PVA), polyacrylamide (PAM), and polyacrylic acid (PAA) [18,19,20]. PVA is a well-established polymer matrix due to its easy formation of films, and it is a non-toxic polymer [21], while the addition of PAA to develop a PVA/PAA GPE has demonstrated superior battery performance due to its good water retention and acceptable ionic conductivity [22]. It has been demonstrated that PAA-KOH allows higher cyclability when compared with the PVA-KOH hydrogel [23]. It is worth noting that GPEs have been employed mostly for flexible devices; however, these can be used in grid energy storage. And, as summarized recently, there are few works dealing with PVA and PAA gel polymer electrolytes for ZABs [24].

Further modifications to the GPEs based on the above-mentioned polymers are made by introducing certain fillers. X. Fan et al. [25] reported, for the first time, the incorporation of SiO_2_ nanoparticles into a porous PVA gel polymer electrolyte for ZABs. The hydroxyl groups of SiO_2_ enabled a higher water retention. The authors observed that 5 wt.% of SiO_2_ increased the water uptake from 150% to 233%, and the rechargeability increased by 30%. On the other hand, ZnO nanoparticles have been incorporated into PVA GPEs as they can easily be dissolved in a KOH solution, where zincate ions can act as corrosion inhibitors, and its addition can also be beneficial during the OER, but it can cause a decrease in ionic conductivity [26]. Recently, graphene oxide was added to a PVA/PAA membrane, where GO allowed PVA crystallinity to be decreased due to the formation of hydrogen bonds between functional groups of GO and the polymer chain [27], which caused higher ionic conductivity and better water retention. As observed, further strategies are required to improve the rechargeability of ZABs operated with non-aqueous electrolytes.

In this work, PVA/PAA membranes were synthesized by the solvent casting method and used as gel polymer electrolytes for ZABs. The PVA/PAA membranes were obtained at three different thicknesses (50, 100, and 150 μm), and the best was modified with SiO_2_ nanospheres at three loadings: 0.5, 2, and 5 wt.%. Then, the best GPE was modified with synthesized multi-walled carbon nanotubes at loadings of 0.1, 1, and 2 wt.% to obtain, for the first time, a PVA/PAA-SiO_2_-CNT gel polymer electrolyte for ZABs. Physicochemical properties like water uptake, ionic conductivity, and ion-exchange capability were determined. In the ZAB, polarization and power density curves were obtained as well as galvanostatic discharge/charge cycling curves and stability discharge curves at different demanding values. Finally, post-mortem tests conducted with scanning electron microscopy were employed to highlight the advantages of using SiO_2_ + CNTs as fillers. 

## 2. Results and Discussion

### Physicochemical Results

**Figure 1:**
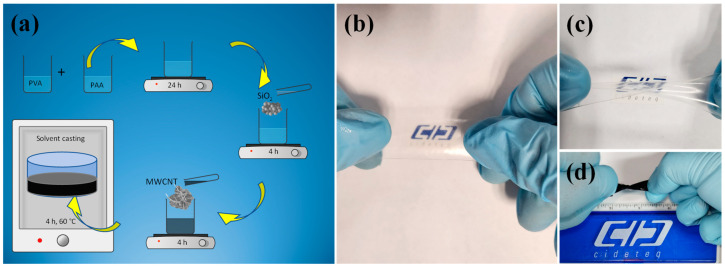
(**a**) Schematic representation of the synthesis of the PVA/PAA membrane modified with silicon dioxide and multi-walled carbon nanotubes. Photographs of (**b**) PVA/PAA membrane, (**c**) PVA/PAA-SiO_2_ 0.5 wt.%, and (**d**) PVA/PAA-SiO_2_-CNT (1 wt.%).

The schematic representation of the methodology to obtain PVA/PAA membranes is illustrated in Figure 1a. The PVA and PAA were dissolved (using an ultrasonic bath) separately in deionized water, and then they were mixed in a 1:1 mass ratio for 24 h under magnetic agitation. Later, the solution was poured into a glass dish, and the membrane was obtained at room temperature. Later, it was placed in an oven at 70 °C for 4 h. A similar procedure was used for the modification with SiO_2_ and CNTs as observed in the figure. The PVA/PAA membranes with thicknesses of 50, 100, and 150 µm were obtained by modulating the poured volume, and all the membranes were translucent as shown in Figure 1b. The PVA/PAA SiO_2_ 0.5 wt.% presented good rolling and stretching capability, as observed in Figure 1c. Nonetheless, with the incorporation of CNT, the membrane turned black, while it only maintained the rolling capability (Figure 1d).

The SEM images in Figure S1a,b reveal that the SiO_2_ particles have a spherical shape with a narrow size distribution, ranging from 80 to 178 nm, and an average diameter of approximately 110 ± 10 nm. The high-magnification image (Figure S1b) highlights the smooth surface characteristic of SiO_2_ nanospheres produced by the Stöber method, confirming the uniformity and high quality of the synthesized particles. The X-ray diffraction (XRD) results for the SiO_2_ sub-microspheres, as depicted in Figure S1c, reveal a broad, diffuse peak centered around 22° to 24° 2θ. This broad peak is indicative of the predominantly amorphous nature of the SiO_2_, which is consistent with the typical XRD profile for Stöber-synthesized SiO_2_ [28]. The elemental analysis obtained via EDS, as illustrated in Figure S1d, corroborates that the spheres were composed exclusively of Si and O with the expected stoichiometry of SiO_2_. The SEM images in Figure S2 demonstrate the formation of carbon nanotubes. The length of this material was in the range of tens of micrometers, as observed in Figure S2a. At a higher magnification (Figure S2b), it is clearly observed that despite washing with nitric acid, some remnants of iron were present in the form of hemispherical particles with nanometric sizes. In Figure S3c, it can be seen that the bright spots observed in Figure S2a,b correspond to the edges of the nanotubes. And, at higher magnifications, it is observed that the carbon nanotubes presented nanometric diameters, finding an average value of 95 ± 7 nm. The XRD pattern of carbon nanotubes (Figure S3a) presented only a sharp peak at 26° 2θ, attributed to the (002) peak of graphite (crystalline card #96-101-1061), confirming the formation of carbon nanotubes in a multi-walled structure [29]. The Raman spectrum (Figure S3b) presented three peaks at 1350, 1575, and 2700 cm^−1^, attributed to the D, G, and G’ bands, where the D band arises from the disorder in the carbon structure and the G band from the high crystallinity of graphitic structures in CNTs. The I_D_/I_G_ gives the degree of disorder in the CNTs; higher ratios than the unit suggest an abundance of surface defects as in this case, where the CNTs presented a ratio of 1.45.

**Figure 2:**
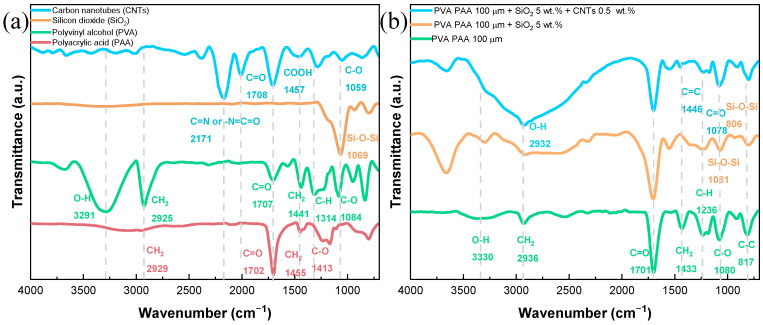
FTIR spectra of (**a**) the individual components of the PVA-PAA membranes and (**b**) the PVA-PAA membrane after modification with SiO_2_ and SiO_2_ + CNT.

The FTIR spectra depicted in Figure 2, spanning the range 700–4000 cm^−1^, reveal significant insights into the chemical structure of the individual components (CNTs, SiO_2_, PVA, and PAA) as well as their interactions within the PVA-PAA gel polymer electrolytes, both before and after modification with SiO_2_ and CNTs. In the spectra of individual components shown in Figure 2a, the FTIR spectra of carbon nanotubes (CNTs) show that a band around 1081 cm^−1^ is attributed to the C–O stretching vibration, signifying the presence of oxygen-containing functional groups [30]. The peak at approximately 1522–1641 cm^−1^ is associated with the sp^2^ hybridized carbon atoms within the graphene-like structure of CNTs, reflecting their intrinsic carbon network; this band may also be indicative of the presence of certain oxygen-containing functional groups, such as carbonyl groups (C=O) associated with surface oxidation [31]. Additionally, the peak observed in the 2180–2000 cm^−1^ region is typically associated with the stretching vibrations of carbon–nitrogen triple bonds (C≡N) or with the presence of isocyanate (–N=C=O) groups. This signal can also be indicative of the presence of specific functional groups introduced during the CNT synthesis or post-synthesis treatment processes. The pronounced peak at 1717 cm^−1^ corresponds to the C=O stretching of carboxylic acid groups, confirming the presence of carboxyl functionalities on the CNTs’ surface [32]. Notably, the absence of signals in the region of 3500–3200 cm^−1^ suggests that O–H stretching vibrations are minimal or not significant, indicating a lack of significant hydroxyl group content on the CNTs [30]. The SiO_2_ spectrum reveals peaks at approximately 1080 cm^−1^ and 800 cm^−1^, corresponding to the asymmetric and symmetric stretching of Si–O–Si bonds. The absence of a peak around 3400 cm^−1^ indicates the hydrophobic nature of SiO_2_, due to the lack of O–H stretching vibrations [30]. 

For the PVA spectrum, key peaks are observed at 3291 cm^−1^ (O–H stretching), 2917 cm^−1^ (CH_2_ asymmetric stretching), 1690 cm^−1^ (C=O stretching), 1425 cm^−1^ (CH_2_ bending), 1324 cm^−1^ (C–H deformation), 1080 cm^−1^ (C–O stretching), and 839 cm^−1^ (C–C stretching). These signals reflect the diverse functional groups and structural characteristics of PVA, including hydroxyl groups, methylene groups, and acetyl groups [33,34,35,36]. The FTIR spectrum of PAA highlights significant peaks at 2949 cm^−1^ (CH_2_ stretching), 1695 cm^−1^ (carboxylate C=O stretching), 1446 cm^−1^ (CH_2_ bending), and 1417 cm^−1^ (C–O stretching within the carboxylic acid group), which indicate the presence of carboxyl groups and related functionalities [37,38].

The FTIR spectrum of the PVA-PAA membrane (Figure 2b) exhibits a combination of features from both individual components. The O–H stretching band at 3330 cm^−1^, characteristic of pure PVA, shifts to a higher wavenumber and becomes broader and weaker in the presence of PAA. This change suggests that the hydrogen bonds in PVA are replaced by stronger hydrogen bonding interactions between PVA and PAA, leading to the formation of a more cohesive polymer network. As a result, the O–H band nearly merges with the broad COOH band from PAA, reflecting the successful integration of the two polymers [39]. Peaks around 2917 cm^−1^ for CH_2_ asymmetric stretching, 1690 cm^−1^ for carbonyl (C=O) stretching from PVA, and 1446 cm^−1^ and 1417 cm^−1^ for CH_2_ bending and carboxylate group vibrations from PAA should be evident. Complementary peaks are likely to appear around 1081 cm^−1^ and 839 cm^−1^, corresponding to C–O stretching and C–C stretching vibrations, respectively. The overall spectrum reflects the successful integration of both polymers, with overlapping peaks indicating the interactions between PVA and PAA.

The FTIR spectrum of the PVA-PAA membrane modified with 5 wt.% SiO_2_ shows notable changes, with weaker peaks appearing around 1080 cm^−1^ and 800 cm^−1^, corresponding to the Si–O–Si asymmetric and symmetric stretching vibrations. The broad band around 3275 cm^−1^ for O–H stretching might shift or change in intensity due to the interaction between SiO_2_ and the hydroxyl groups in the PVA-PAA matrix. The presence of SiO_2_ could also influence the intensity and position of other peaks, potentially altering the C–O stretching vibrations around 1081 cm^−1^ and the overall spectral profile, as the SiO_2_ particles may interact with the polymer matrix and modify the local environment of the functional groups. Finally, in the FTIR spectrum analysis of the PVA-PAA blend modified with 5 wt.% SiO_2_ and further enhanced with 0.5 wt.% CNTs, several noteworthy spectral features provide insights into the structural and chemical interactions within the gel polymer matrix. The band observed at 1620 cm^−1^ is initially present in the spectrum of pure PVA, which corresponds to the C=C stretching vibrations of the PVA polymer chains. This band is notably reduced in intensity when PVA is blended with PAA, suggesting that the PAA component affects the vibrational environment of the PVA matrix. The incorporation of 5 wt.% SiO_2_ into the PVA-PAA blend leads to the reappearance of this band with increased intensity, indicating that SiO_2_ enhances the structural interactions within the polymer matrix. The presence of 0.5 wt.% CNTs further intensifies this band, which can be attributed to the interactions between the CNTs and the polymer matrix that modify the vibrational characteristics of the PVA component. The band at 2932 cm^−1^, associated with the O–H stretching vibrations, shows significant broadening and an increase in intensity with the addition of CNTs. This broadening is attributed to the disruption of the hydrogen bonding network within the polymer matrix caused by the incorporation of CNTs. The CNTs likely alter the local environment around the O–H groups, enhancing the band and reflecting changes in the water retention capacity and structural dynamics of the gel polymer electrolyte. Furthermore, the presence of CNTs also influences the intensity and positioning of other spectral features. Notably, it may induce shifts or broadening in the O–H stretching band within the range of 2900–3280 cm^−1^ and affect the C–O stretching vibrations around 1081 cm^−1^. These changes reflect the complex interactions between the CNTs and the polymer matrix, underscoring the CNTs’ role in modifying the material’s electrochemical properties and performance. 

**Figure 3:**
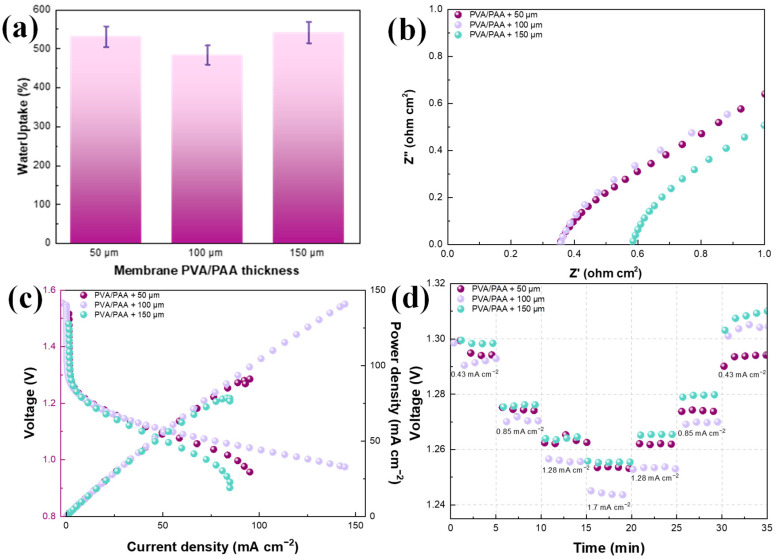
(**a**) Water uptake for PVA-PAA membranes with different thicknesses, (**b**) Nyquist plot at the open-circuit voltage of ZABs assembled with PVA-PVAA membranes, (**c**) polarization and power density curves, and (**d**) stability curves at different demanding current densities.

The obtained PVA/PAA membranes at different thicknesses were analyzed by means of water uptake in 6 M KOH (Figure 3a). As observed, no significant changes were found, and it is expected that since the nature of the membranes was the same, only the thickness changed, and the average water uptake presented by these membranes was 530%, which was higher than that presented by a porous PVA-6 M KOH (four times) [25], PVA/PAA soaked in 1 M NaOH [39], and an internally interconnected porous PVA/PAA membrane (66%) [40]. To further determine the effect of the membrane thickness, these were soaked in the KOH solution and the ZABs were assembled with a commercially available Zn electrode and Pt/C + IrO_2_/C as the catalyst for the air electrode. The Nyquist plots to analyze battery resistance at the open-circuit voltage (OCV, Figure 3b) showed that real impedances below 0.4 Ω cm^2^ were found for those batteries assembled with PVA/PAA membranes with 50 and 100 μm thicknesses. Because the main components were kept similar, only two aspects could affect the inner resistance: the ionic conductivity and the resistance from the electrode separation. Thus, it was found that no significant changes were observed in battery resistance between these two thicknesses, and in fact, the resistance was similar to that reported for an internally interconnected porous PVA/PAA membrane [40]. The increase in the membrane thickness to 150 μm caused an increase in the real impedance to near 0.6 Ω cm^2^, and because of the resemblance in ionic conductivity, this can be attributed to the resistance from increasing the electrode separation. 

The polarization curves displayed in Figure 3c show differences in battery performance with the use of PVA/PAA membranes with different thicknesses. The OCV ranged from 1.48 V (PVA/PAA 150 μm) to 1.56 V (PVA/PAA 100 μm), where the highest, as observed, was found with a 100 μm thickness. The polarization behavior from the OCV to 1.25 V was similar because the catalysts were the same in the three ZABs. From 1.3 to 1.15 V, slight differences were found in the ohmic region, and they were related to the thickness, showing that the decrease in ohmic losses was directly proportional to the decrease in the thickness. In addition, the most noticeable effect was on the zone of limitations by mass transport (from 1.1 to 0.95 V), where the increase in the electrode separation at 150 μm (from the membrane thickness) limited the battery performance. The use of the membrane at 50 μm was not enough to maintain adequate ion mobility, because water evaporation was taking place due to the heat released during the electron transfer in the electrochemical reactions. Thus, the best performance was obtained with the PVA/PAA 100 μm, where at 1 V, the current density was 145 mA cm^−2^, which is almost twice that obtained for the membrane of 150 μm thickness. The batteries were discharged at different current densities to evaluate the required electrical work (voltage), and then the demand was decreased to the initial value (Figure 3d) to analyze the differences between the initial and final conditions. In general, no significant differences were observed during the comparison between the initial and final conditions, and only the increase in the voltage to provide 0.43 mA cm^−2^ at the end could be a result of the catalyst activation process. Consequently, the PVA/PAA membrane with 100 μm thickness was selected for further modifications. 

**Figure 4:**
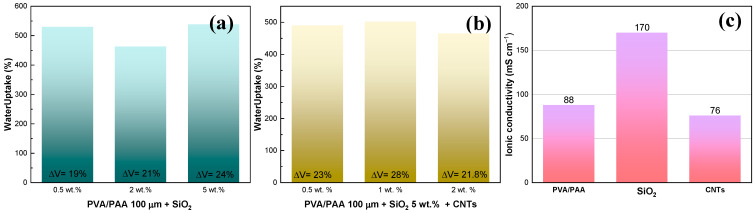
(**a**) Water uptake with the incorporation of SiO_2_ at different contents, (**b**) water uptake as a function of CNT content fixing the SiO_2_ concentration, and (**c**) ionic conductivity, with 5 wt.% SiO_2_, and with 5 wt.% SiO_2_ + 1 wt.% CNT. Volumetric changes during water uptakes are presented inset the figures.

The incorporation of SiO_2_ nanospheres into the PVA/PAA 100 μm membrane slightly increased the water uptake from 465% to 520% at 0.5 wt.%, and to 525% at 5 wt.% (Figure 4a). The decrease in water uptake from 465 to 460% with 2 wt.% SiO_2_ could be related to particle agglomeration, while the lack of larger increments could be related to the low hydrophilic behavior of SiO_2_ (Figure S1a). It is worth noting that SiO_2_ was added not only as a water retainer, but also as a plasticizer and to improve the chemical stability of the membrane [25], while carbon nanotubes were added to improve the mechanical stability of the membrane and to enhance the ion mobility due to the creation of internal pores [27]. In the case of the PVA/PAA-SiO_2_ 5 wt.% (as representative material) membranes with different contents of carbon nanotubes (Figure 4b), it was found that the addition of these did not have an influence on the water uptake, and the slight decrease in % in contrast to the membrane without CNTs (Figure 4a) could be related to the possible formation of domains with nanoparticle aggregates, which can be confirmed with the ionic conductivity measurements (Figure 4c), which showed that CNT decreased the conductivity from 88 mS cm^−1^ for the PVA/PAA membrane to 76 mS cm^−1^. The highest value of ionic conductivity was displayed by the PVA/PAA membrane with 5 wt.% SiO_2_ (170 mS cm^−1^).

**Figure 5:**
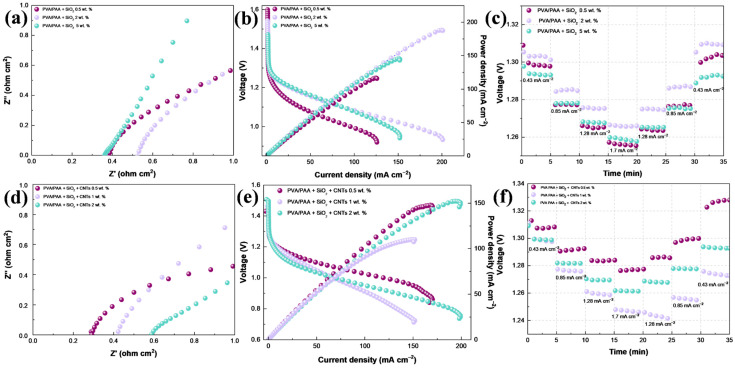
(**a**) Nyquist plot, (**b**) polarization and power density curves, and (**c**) stability curves at different demanding current densities for the ZAB assembled with PVA-PAA membranes modified with SiO_2_ at different contents. (**d**) Nyquist plot, (**e**) polarization and power density curves, and (**f**) stability curves at different demanding current densities for the ZAB assembled with PVA-PAA membranes modified with 5 wt.% of SiO_2_, varying the content of CNTs.

As previously mentioned, the lower water uptake in the PVA/PAA membrane with 2 wt.% SiO_2_ could be related to particle agglomeration, and, interestingly, the battery assembled with this membrane presented the highest battery resistance (Figure 5a). Additionally, the other two batteries displayed a similar behavior, in which higher water uptake percentages enabled lower battery resistances, which could be related to better interaction with the electrodes due to wettability. In the polarization curves (Figure 5b), it was found that despite the membrane with 2 wt.% SiO_2_ having higher battery resistance, it was the membrane presenting a higher current density (200 mA cm^−2^), and thus, a higher power density (190 mW cm^−2^). The SiO_2_ incorporation at 0.5 wt.% showed mass transport limitations presenting lower current densities even compared to the ZAB with the PVA/PAA and without SiO_2_ (Figure 5c). The batteries with 2 and 5 wt.% presented identical behavior in the ohmic region and the beginning of the mass transport region, displaying the same current and power density from 1.3 V to 1.08 V; perhaps due to the higher content of SiO_2_, the performance of the 2 wt.% battery was greater. Additionally, no major changes were observed in the stability curve presented in Figure 5c. Despite the ZAB with 2 wt.% SiO_2_ displaying higher performance, herein we used 5 wt.% SiO_2_ for further tests since stability time has been found to increase with SiO_2_ content [25]. 

The Nyquist plots for the ZABs assembled with CNTs (Figure 5d) indicated that the battery resistance increased with the CNT content, which is expected since carbon nanotubes do not have an intrinsic contribution to ionic conductivity, as shown in Figure 4c. The battery performance did not follow any specific trend, which could be caused by deficiencies in the dispersion of carbon nanotubes during the curing of the membrane or other effects, as is discussed later. This was confirmed during mechanical tests; the use of CNTs at 0.5 wt.% maintained the folding characteristic of the PVA/PAA membrane (Figure S4a), while higher concentrations (1 wt.%) induced a loss of stretchability (Figure S4b). Lower ohmic resistances were found for the ZAB employing 0.5 wt.% CNTs (Figure 5e), achieving the highest current density at a higher voltage of 0.91 V (at 1 wt.%, it was found at 0.8 V, and at 2 wt.% at 0.82 V). Thus, it achieved the maximum power density (147 mW cm^−2^) at a lower overpotential from the OCV (550 mV vs. 730 mV for the ZAB with 2 wt.%). The battery assembled with 2 wt.% presented slightly higher power density than that with 0.5 wt.%, but as mentioned, it was achieved at a larger overpotential. In addition, this battery and the battery operated with 1 wt.% CNTs presented poor stability due to the ending voltage being lower than that at the beginning when demanding 0.43 mA cm^−2^ (Figure 5f). On the other hand, the battery operated with the addition of 0.5 wt.% CNTs displayed excellent stability.

**Figure 6:**
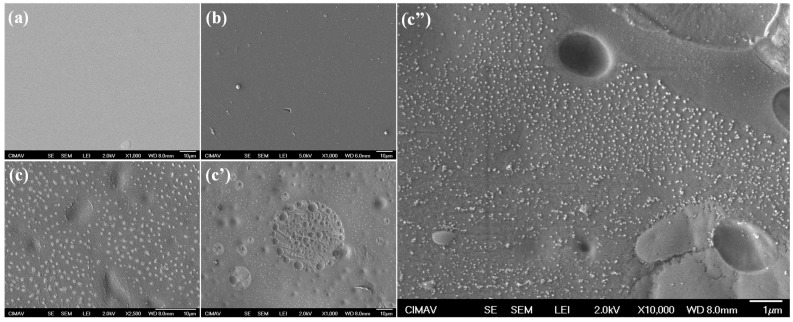
SEM micrographs for (**a**) the PVA/PAA membrane, (**b**) the PVA/PAA membrane with 5 wt.% SiO_2_, and (**c**,**c’**,**c’’**) the PVA/PAA membrane with 5 wt.% SiO_2_ and 0.5 wt.% CNTs at different magnifications.

The SEM micrographs (Figure 6) of the three membranes (PVA/PAA, PVA/PAA-SiO_2_, and PVA/PAA-SiO_2_-CNTs) were analyzed to elucidate the morphological factors contributing to the observed differences in battery performance (Figure 5) between the systems containing only SiO_2_ and those incorporating CNTs. The PVA/PAA membrane shows a smooth, defect-free surface (Figure 6a). The addition of SiO_2_ does not visibly alter the overall appearance of the membrane matrix; however, at the microscale, some micrometric SiO_2_ spheres are present on the surface (Figure 6b). The incorporation of CNTs, on the other hand, significantly modified the membrane morphology by introducing porosity and surface defects, as observed in Figure 6c,c’c’’. Although this inclusion did not substantially impact water retention, it decreased ionic conductivity. The increased porosity and surface irregularities likely enhanced the surface area, potentially contributing to the higher power density observed. Moreover, the nanometric and micrometric features on the surface suggest that CNTs may facilitate the migration of SiO_2_ particles to the membrane surface, which could explain the reduction in ionic conductivity. These morphological changes underscore the complex interactions within the composite membranes and their significant influence on battery performance.

**Figure 7:**
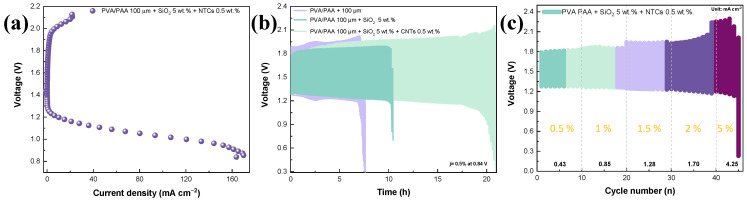
(**a**) Charge and discharge curve for the ZAB assembled using PVA-PAA with optimized SiO_2_ and CNT content. (**b**) Charge/discharge cycles for the ZAB assembled with three gel polymer electrolytes: PVA-PAA, PVA-PAA + SiO_2_, and PVA-PAA + SiO_2_ + CNT. (**c**) Charge/discharge cycles using different current density values for the ZAB assembled with the optimized PVA-PAA + SiO_2_ + CNT gel polymer electrolyte. The percentages presented in incises (**b**,**c**) represent the demanded current densities considering the maximum current density at 0.8 V.

The effect of introducing SiO_2_ and CNTs to the PVA/PAA membrane was also studied in terms of the rechargeability of the ZABs (Figure 7). The charge/discharge curves for the PVA/PAA-SiO_2_-CNTs are presented in Figure 7a as representative material. The discharge process was previously discussed, while the charge process indicates that a polarization occurred from the OCV to 1.9 V. Then, the charging process (involving the Zn reduction and the OER) took place, and a plateau was found at 20 mA cm^−2^, followed by electrode passivation. The voltage gap varied from 0.5 to 0.9 V for those currents from near zero to 20 mA cm^−2^. When demanding 0.5% of the maximum current density (0.43 mA cm^−2^), the charge/discharge cycles presented initial voltage gaps of 493, 513, and 617 mV for the ZABs operated with the PVA/PAA-SiO_2_-CNTs, the PVA/PAA-SiO_2_, and the PVA/PAA membrane, respectively (Figure 7b). This result indicates that the most favorable gel polymer electrolyte is that obtained by integrating SiO_2_ and CNTs. The initial round-trip efficiency increased from 67.2% for the unmodified PVA/PAA membrane to 73.7% with the PVA/PAA-SiO_2_-CNTs. The rechargeability of the ZAB with PVA/PAA rapidly decreased after a few hours, showing that the discharge is the limiting step, which is not unexpected due to the huge instability of Pt/C in alkaline conditions [41,42]. The introduction of SiO_2_ increased the rechargeability, where the battery remained functional after almost 10 h. And, despite presenting a higher voltage gap, the ZAB operated with the PVA/PAA-SiO_2_-CNTs displayed twice the durability than that operated only with SiO_2_ as a filler, and at eighteen hours, the round-trip efficiency remained acceptable (64.1%). In addition, it is worth mentioning that a higher rechargeability could be achieved by modulating the interface through the usage of highly stable bifunctional electrocatalysts. Finally, the ZAB operated with the VA/PAA-SiO_2_-CNTs displayed the capability to operate at higher current densities, being stable at percentages of current densities from 0.5 to 5% with regard to the maximum current density value (Figure 7c).

**Figure 8:**
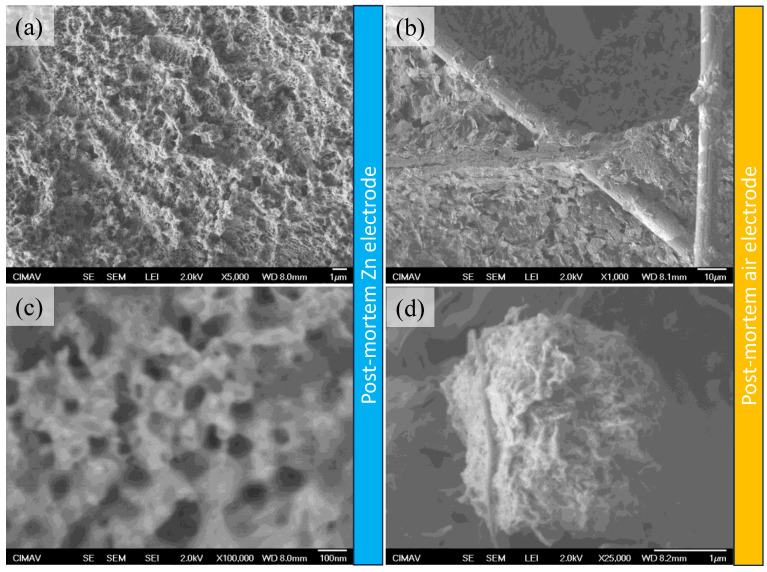
SEM micrographs for the Zn (**a**,**c**) and air electrodes (**b**,**d**) after the charge/discharge cycles for the ZAB assembled with the PVA-PAA + SiO_2_ GPE.

**Figure 9:**
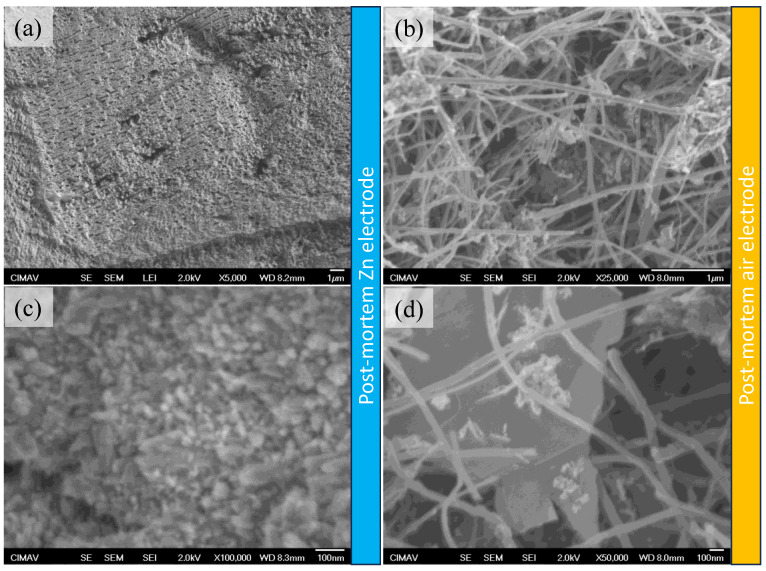
SEM micrographs for the Zn (**a**,**c**) and air electrodes (**b**,**d**) after the charge/discharge cycles for the ZAB assembled with the PVA-PAA + SiO_2_ + CNT GPE.

After the galvanostatic charge/discharge (GCD) curves, post-mortem tests were conducted through the acquisition of SEM images from the Zn anode and from the air electrode for those materials from the ZAB assembled with the PVA/PAA-SiO_2_ (Figure 8) and the PVA/PAA-SiO_2_-CNT membrane (Figure 9). The Zn electrode transformed from a smooth to a porous surface. In Figure 8c, the presence of pores at the nanometric scale is observed, and the formation of the structures may be related to the presence of gas bubbles trapped during discharge, which serves as a template for hierarchical porous structures. In the literature, there is a synthesis method called dynamic electrodeposition on bubbles [43], in which similar architectures are reported. Consequently, two aspects can be highlighted from these SEM images: (i) the use of a GPE based on PVA/PAA-SiO_2_ can allow the synthesis of 3D porous structures of Zn during charge, which can enhance the battery durability, and (ii) this phenomenon of trapping gas bubbles avoids the formation of Zn dendrites. In the case of the air electrode (Figure 8b,d), the SEM micrographs revealed that the tubular structures of Sigracet carbon paper were mostly naked, while micrometric aggregates were also observed. In this manner, the loss of material from the air electrode and the electrocatalyst agglomeration can be found as a consequence of the failure in the rechargeability test presented in Figure 7.

In the case of the Zn anode from the ZAB tested with the PVA/PAA-SiO_2_-CNT gel polymer electrolyte (Figure 9), it was found that it maintained a smoother surface (Figure 9a) than the previously discussed Zn electrode, while some nanometric pores were still observed. It was observed at a higher magnification (Figure 9c) that a nanometric deposit was obtained after the rechargeability test, suggesting that this gel polymer electrolyte not only promoted a decrease in the HER, but also avoided the formation of Zn dendrites, while a deposit with a finer grain could be obtained. In the case of the air electrode (Figure 9b,d), the presence of carbon nanotubes on the air electrode surface was observed, which could induce a better interaction of the electrode/electrolyte interface. However, the presence of agglomerations is also found, which indicates that the instability of the bifunctional electrocatalyst was the main reason for the loss of rechargeability, rather than the electrolyte. This is highly important, since for this GPE, a higher rechargeability can be achieved by using Pt-free electrocatalysts. 

## 3. Conclusions

This study highlights the successful fabrication and characterization of PVA/PAA-based membranes and their modifications with SiO_2_ and CNTs, providing significant insights into their structural, chemical, and electrochemical properties. This investigation emphasizes the significant impact of membrane thickness, with controlled values of 50, 100, and 150 µm, on internal resistance and electrochemical performance. The membranes with a thickness of 100 µm achieved an optimal balance between ionic conductivity and electrode resistance, resulting in superior battery performance with a current density of 145 mA cm^−2^ at 1 V. The incorporation of SiO_2_ into PVA/PAA membranes enhanced water retention capacity and improved chemical stability, with an optimal concentration of 5 wt.% demonstrating the greatest increase in ionic conductivity (170 mS cm^−1^), while CNTs, despite their influence on membrane color and a slight reduction in ionic conductivity, contributed to mechanical stability. However, CNTs at low concentrations (0.5 wt.%) introduced porosity to the membrane, while decreasing the HER and formation of Zn dendrites, providing improved operational stability and enhancing overall battery rechargeability. These findings underscore the critical role of both membrane thickness and composition in optimizing battery efficiency and stability. Specifically, membranes modified with 5 wt.% SiO_2_ and 0.5 wt.% CNTs demonstrated the best balance of performance and stability. This study offers a refined understanding of polymer membrane technologies, setting a precedent for future advancements in energy storage applications and highlighting the potential of these materials for improved battery systems. The limitations of this study are related to the dispersion of the components during the formulation of the casting solution, and further improvements like using a high-power ultrasonic probe are undergoing.

## 4. Materials and Methods

### 4.1. Synthesis of PVA/PAA Membranes

In a typical synthesis, poly(vinyl alcohol) (Sigma-Aldrich, St. Louis, MO, USA, MW: 146,000) and poly(acrylic acid) (Sigma-Aldrich, MW: 450,000) were weighed in a 1:1 ratio and dissolved individually in 5 mL deionized water assisted by ultrasonic agitation (1 h). Then, the solutions were mixed in a 25 mL baker and maintained at room temperature and under magnetic agitation during 24 h to ensure a homogeneous solution. Then, certain volumes were verted in glass dishes (6 cm in diameter) to modify the film thickness, and trapped air was removed at room temperature in an oven with a vacuum. The membranes were formed in air, and then heated at 70 °C for 4 h. The further modification with SiO_2_ and CNTs was performed similarly; after 24 h agitation, certain amounts of SiO_2_ and CNTs were added to the oven and the agitation was continued for another 4 h. The further procedure was maintained as previously mentioned. 

### 4.2. Synthesis of SiO_2_ and CNTs

The SiO_2_ nanoparticles were obtained by the Stöber method. Tetraethyl orthosilicate (TEOS, Sigma-Aldrich, ≥99.0%) was used as the precursor, ethanol (J.T. Baker, Phillipsburg, NJ, USA, >99%) as the reaction medium, and deionized water and ammonium hydroxide (Sigma-Aldrich, ACS reagent, 28–30%) as the initiators. The reaction was performed in a round-bottom flask submerged in an oil bath and connected to a condenser. First, 50 mL ethanol were placed in the flask, followed by 5 mL deionized water and 2 mL TEOS (0.18 M), and the system was maintained under magnetic agitation, while the temperature was raised to 60 °C. Then, 0.5 mL ammonium hydroxide (0.71 M) weas added, and the reaction was maintained for 12 h. Then, the milky solution was washed two times with ethanol and two times with acetone. The resulting powder was recovered with centrifugation and heated at 100 °C for 2 h to eliminate solvent remnants. CNTs were prepared as reported elsewhere [44]. The resulting CNTs were purified in a 15 M HNO_3_ solution (Sigma-Aldrich, ACS reagent, 70%) at 130 °C for 12 h under magnetic agitation. Then, the powder was washed several times until the pH was near 7, and then the powder was dried at 70 °C overnight.

### 4.3. Physicochemical and Electrochemical Characterization

SEM micrographs were obtained using a field-emission scanning electron microscope (Jeol JSM7401F, Boston, MA, USA), and EDS analysis was performed by a EDAX coupled to the SEM apparatus. Raman and FTIR spectroscopies were performed using a Horiba XplorA PLUS (Longjumeau, France) and a Nicole Magna-IR spectrometer model 750 (Markham, ON, Canada). X-ray diffraction patterns were obtained in a Bruker D8 Advance diffractometer (Billerica, MA, USA). Water uptakes and ionic conductivities were determined as reported elsewhere [45].

The ZAB was constructed by modifying a previously reported battery for aqueous electrolytes [46], where the reservoir for the aqueous electrolyte was removed, and instead, the membrane was placed acting as a GPE and sealer. A silicon film (with similar dimensions of the membrane) was added as a sealer. A Zn plate (Kolamoon, Hefei, Anhui, China, 3 mm in thickness, 99.9%) was used as the Zn electrode, a Sigracet 25 BC (FuelCellStore, Bryan, TX, USA, 235 μm) was used as the air electrode, and benchmarked Pt/C and IrO_2_/C (FuelCellStore, Bryan, TX, USA, 20 wt.%) were used as catalysts. These were mixed in a 1:1 mass ratio and deposited by the hot spray technique until a mass loading of 1 mg cm^−2^ was achieved. A Gamry Reference 3000 Potentiostat/Galvanostat was used for the battery tests, while the stability tests were conducted for 5 min by chronoamperometry at current densities of 0.43, 0.85, 1.28, and 1.7 mA cm^−2^. The galvanostatic charge/discharge cycles were obtained at 0.43 mA cm^−2^ for 5 min per half-cycle (cycle duration: 10 min). The Nyquist plots were obtained at the OCV in a frequency range from 100,000 to 0.1 Hz. 

## Data Availability

Dataset available on request from the authors.

## Supplementary Materials

The following supporting information can be downloaded at: https://www.mdpi.com/article/10.3390/gels10090587/s1, Figure S1: a,b) SEM micrographs of the synthesized SiO_2_ nanoparticles, c) X-ray diffraction pattern and d) EDS analysis; Figure S2: SEM micrographs of the synthetized multi-walled carbon nanotubes at different magnifications: a) 5000×, b) 10,000×, c) 25,000× and d) 50,000×; Figure S3: a) X-ray diffraction pattern and b) Raman spectrum of multi-walled carbon nanotubes; Figure S4: Photographs of the PVA/PAA-SiO_2_-CNTs under different mechanical efforts.
