# Supplementary material for: Poly(Vinyl Alcohol)/Poly(Acrylic Acid) Gel Polymer Electrolyte Modified with Multi-Walled Carbon Nanotubes and SiO2 Nanospheres to Increase Rechargeability of Zn–Air Batteries
Source: Gels. 2024 Sep 12;10(9):587. doi: 10.3390/gels10090587 (PMC11431819; doi:10.3390/gels10090587)
Supplement: Supplementary file 1 [file gels-10-00587-s001.zip › gels-3185411-supplementary.pdf]

Supplementary information

Poly(Vinyl Alcohol)/Poly(Acrylic Acid) Gel Polymer Electrolyte Modified with Multi-Walled Carbon Nanotubes and SiO_2 Nanospheres to Increase Rechargeability of Zn–Air Batteries

Lucia Díaz-Patiño [1], Minerva Guerra-Balcázar [2], Lorena Álvarez-Contreras [3,*] and Noé Arjona [1,*]

1 Centro de Investigación y Desarrollo Tecnológico en Electroquímica, Sanfandila, Pedro Escobedo,
Querétaro 76703, Mexico; apatino@cideteq.mx
2 División de Investigación y Posgrado, Facultad de Ingeniería, Universidad Autónoma de Querétaro,
Querétaro 76010, Mexico; minerva.guerra@uaq.mx
3 Centro de Investigación en Materiales Avanzados S.C., Complejo Industrial Chihuahua, Chihuahua 31136, Mexico
* Correspondence: lorena.alvarez@cimav.edu.mx (L.Á.-C.); wvelazquez@cideteq.mx (N.A.); Tel.: +52-614-439-1119 (L.Á.-C.); +52-442-211-6000 (ext. 7874) (N.A.)

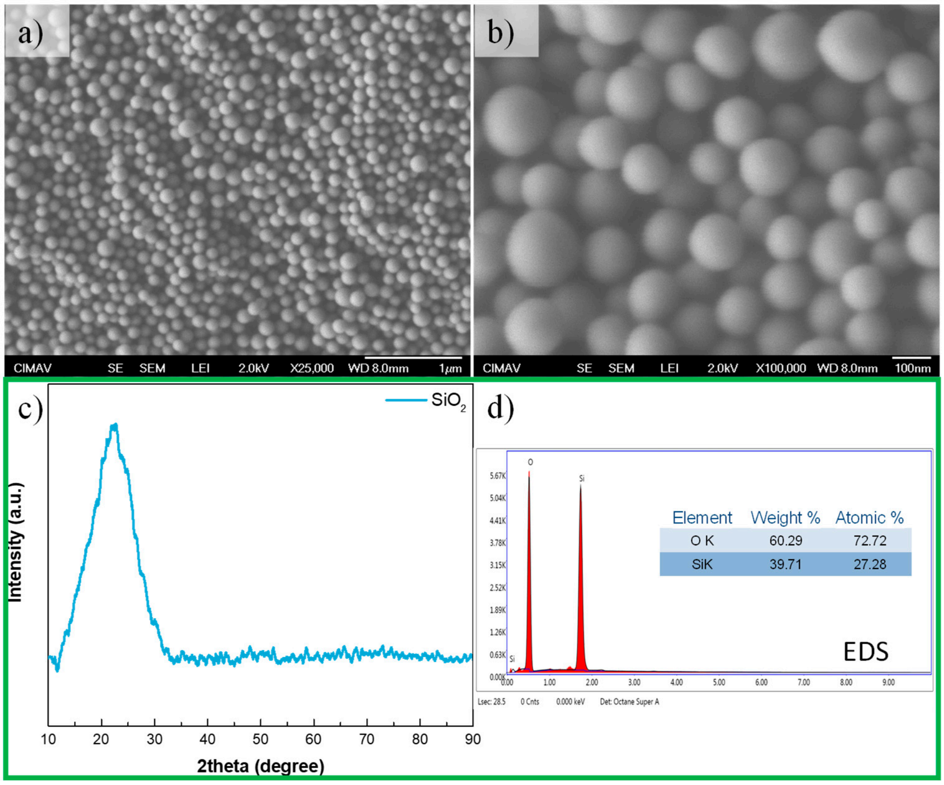

Figure S1. a, b) SEM micrographs of the synthesized SiO_2 nanoparticles, c) X-ray diffraction pattern and d) EDS analysis.

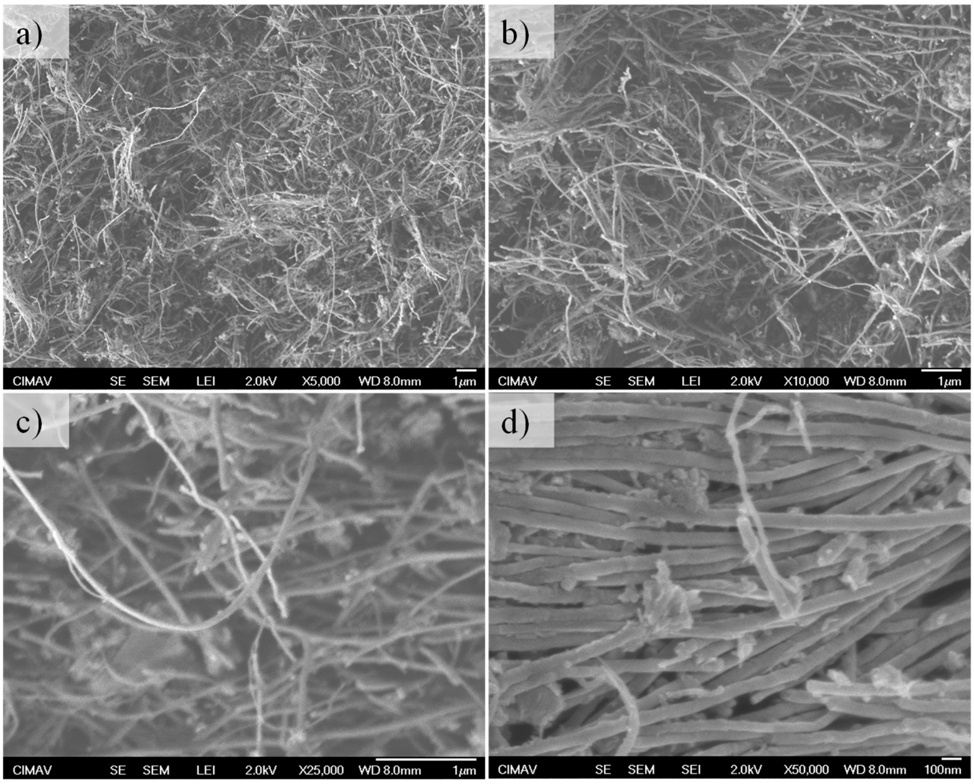

Figure S2. SEM micrographs of the synthetized multi-walled carbon nanotubes at different magnifications: a) 5000x, b) 10000x, c) 25000, and d) 50000x.

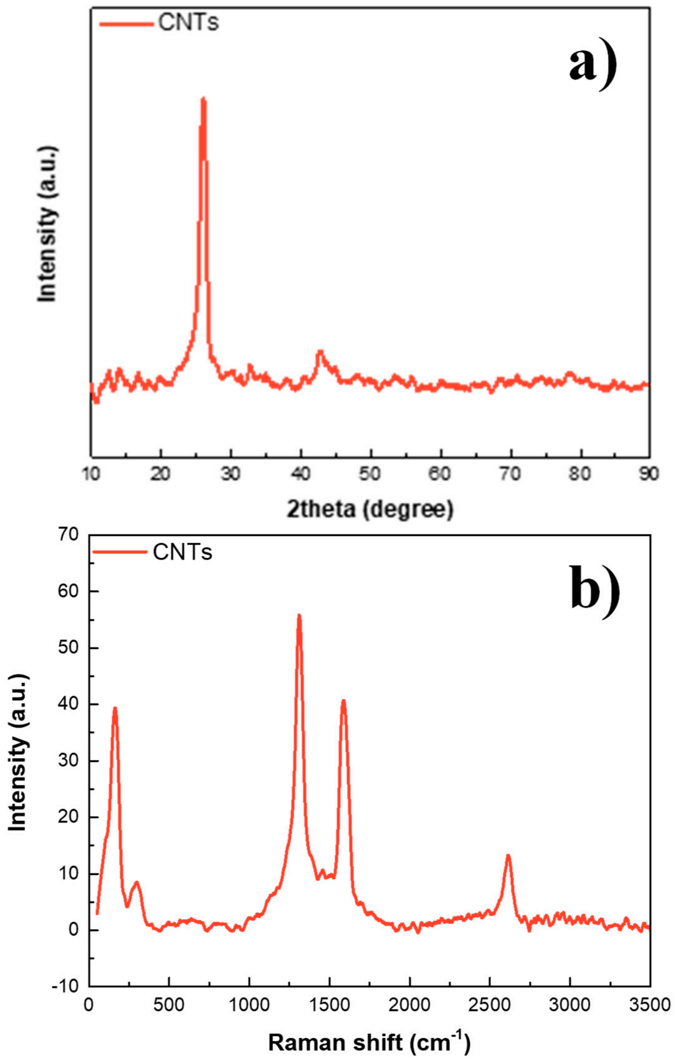

Figure S3. a) X-ray diffraction pattern and b) Raman spectrum of multi-walled carbon nanotubes.

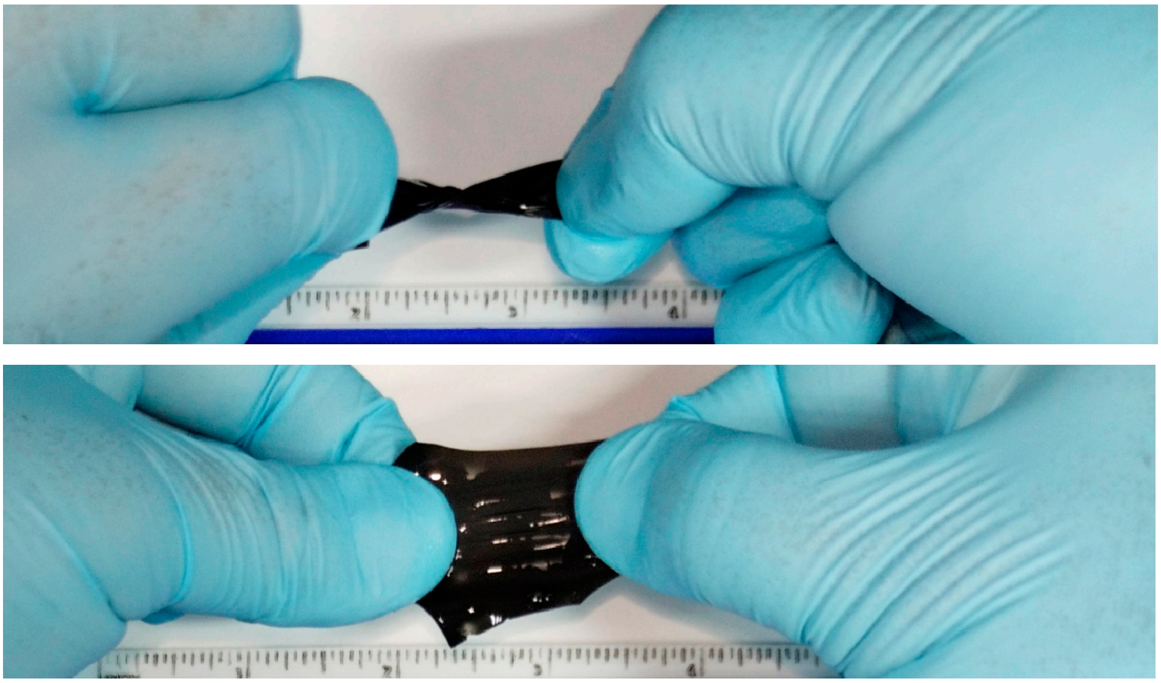

Figure S4. Photographs of the PVA/PAA-SiO_2-CNTs under different mechanical efforts
